# Supplementary material for: Information sharing and deferral option in cybersecurity investment
Source: PLoS One. 2023 Feb 6;18(2):e0281314. doi: 10.1371/journal.pone.0281314 (PMC9901811; doi:10.1371/journal.pone.0281314)
Supplement: S1 Appendix — (DOCX) [file pone.0281314.s001.docx]

Appendix

**Proof of Proposition 1:** $\rho_{1in\_sha}\geq\rho_{2in\_sha}$ and that $\rho_{1in\_sha},\rho_{2in\_sha}$ cannot be positive and less than one at the same time according to the proof of Result3 in Cavusoglu and Raghunathan [32]. We found the following optimization conditions:

$\frac{\partial M_{sha}}{\partial\rho_{1in\_sha}}=\psi_{sha}P_{D}\left( H-C \right)+\left( 1-\psi_{sha} \right)P_{F}(L-C)$ (A1)

$\frac{\partial M_{sha}}{\partial\rho_{2in\_sha}}=\psi_{sha}\left( 1-P_{D} \right)\left( H-C \right)+\left( 1-\psi_{sha} \right)\left( 1-P_{F} \right)(L-C)$ (A2)

$\frac{\partial A_{sha}}{\partial\psi_{sha}}=P_{D}\left( \mu-\beta\rho_{1in\_sha} \right)+\left( 1-P_{D} \right)\left( \mu-\beta\rho_{2in\_sha} \right)$ (A3)

We solve mixed equilibrium strategies.

(a) If ($\rho_{1in\_sha}=1, 0<\rho_{2in\_sha}<1, 0<\psi_{sha}<1$) is an equilibrium, the first-order conditions with respect to $\psi_{sha}$ and $\rho_{2in\_sha}$must be satisfied. Given that $\rho_{1in\_sha}=1$, equating (A2) and (A3) to zero cost savings

$\rho_{2in\_sha}^{*}=\frac{\mu-\beta P_{D}}{\beta\left( 1-P_{D} \right)}$ (A4)

$\psi_{sha}^{*}=\frac{\left( 1-P_{F} \right)\left( C-L \right)}{\left( 1-P_{D} \right)\left( H-C \right)+\left( 1-P_{F} \right)\left( C-L \right)}$ (A5)

Because $0<\rho_{2in\_sha}<1$, we obtain $P_{D}\leq\frac{\mu}{\beta}$.

(b) If ($0<\rho_{1in\_sha}<1, \rho_{2in\_sha}=0, 1<\psi_{sha}<1$) is an equilibrium, then the first-order conditions with respect to $\psi_{sha}$ and $\rho_{1in\_sha}$ must be satisfied. Equating (A1) and (A3) to zero and substituting $\rho_{2}=0$ yield the following:

$\rho_{1in\_sha}^{*}$=$\frac{\mu}{\beta P_{D}}$ (A6)

$\psi_{sha}^{*}=\frac{P_{F}\left( C-L \right)}{P_{D}\left( H-C \right)-P_{F}\left( C-L \right)}$ (A7)

Because of $0<\rho_{1in\_sha}<1$, we obtain $\frac{\mu}{\beta}<P_{D}.$
