# Supplementary material for: Information sharing and deferral option in cybersecurity investment
Source: PLoS One. 2023 Feb 6;18(2):e0281314. doi: 10.1371/journal.pone.0281314 (PMC9901811; doi:10.1371/journal.pone.0281314)
Supplement: Attachment — Submitted filename: PONE-D-22-24920.docx [file pone.0281314.s002.docx]

September 16, 2022

Report PONE-D-22-24920

1. The article provides an interesting analysis of information sharing and the deferral option in security investment.
2. One main contribution is to include the deferral option, which is not much analyzed in the literature.
3. A native reader is needed. The article ignores most rules on singular/plural, when to use “the,” etc.
4. The article should specify that the analysis is about information sharing between firms, not information sharing between attackers.
5. The article considers only one firm, abstracting away that information sharing actually occurs between at least two firms (or between at least two attackers). This limitation should be discussed. Especially, how can the results be realistic when only one firm is analyzed?
6. The first sentence in the abstract says “information security investment strategies.” The article should specify more clearly exactly which number of strategies are available for each player.
7. The deferral option should be defined more clearly. What is being deferred? The reader quickly realizes that the firm’s security investment (singular or plural?) may or may not be deferred. Can information sharing be deferred? Why does the reader have to search forever to find out whether or not information sharing can be deferred?
8. The authors should ensure that all the articles in the reference list, checking one by one, are cited inside the article.
9. The alpha is crucial, and should probably be different for the firm and the attacker.
10. The article’s focus on the deferral option pertains to whether the firm is proactive by investing early, or retroactive by investing later after a deferral. Comparison of the approach and results with the following article seems useful: Hausken, K. (2018), “Proactivity and Retroactivity of Firms and Information Sharing of Hackers,” International Game Theory Review 20, 1, 1750027, doi: 10.1142/S021919891750027X.
11. More generally, comparing the approach and results with the articles in the reference list should be made more thoroughly, accounting for the fact that the article considers only one firm.
12. The weak abstract should be strengthened substantially, listing and discussing the results, emphasizing the contribution relative to the literature, etc. Conclusions can be written without parameters.
