# Supplementary material for: Information sharing and deferral option in cybersecurity investment
Source: PLoS One. 2023 Feb 6;18(2):e0281314. doi: 10.1371/journal.pone.0281314 (PMC9901811; doi:10.1371/journal.pone.0281314)
Supplement: Attachment — Submitted filename: Response to Reviewers.docx [file pone.0281314.s003.docx]

Response to Reviewers

Dear Reviewers:

Thank you for your review, we have amended this article according to your advice. All the question and answer are as follows:

1. The article provides an interesting analysis of information sharing and the deferral option in security investment.

2. One main contribution is to include the deferral option, which is not much analyzed in the literature.

**Answer:** we have added the analysis of the deferral option in the third paragraph of introduction.

3. A native reader is needed. The article ignores most rules on singular/plural, when to use “the,” etc.

**Answer:** we have checked and fixed all the rules on singular/plural, when to use “the,” etc.

4. The article should specify that the analysis is about information sharing between firms, not information sharing between attackers.

5. The article considers only one firm, abstracting away that information sharing actually occurs between at least two firms (or between at least two attackers). This limitation should be discussed. Especially, how can the results be realistic when only one firm is analyzed?

**Answer to question 4 and 5**: This study assumes that firms have joined an industry-specific information-sharing group. No charges are incurred for joining this information-sharing group, providing that a firm is willing to share cybersecurity-related information with the group’s members (i.e., free-riders are excluded from this group). Based on the agreement, all firms report detailed information to the group’s members on their actual cybersecurity breaches and the steps taken to prevent and respond to cybersecurity breaches. Hence, the study constructs a game model between a firm and an attacker; however, the firm can enjoy information sharing. In this study, information sharing occurs between the members of the industry-specific information-sharing group. Additionally, the analysis in this study concerns information sharing between firms, not information sharing between attackers. All of these has been specified in the previous section of the model description.

6. The first sentence in the abstract says “information security investment strategies.” The article should specify more clearly exactly which number of strategies are available for each player.

**Answer:** A firm has three strategies: immediate investment, no investment, and decision-making after deferral. An attacker has only two strategies: intrusion and no intrusion. All of these has been specified in the previous section of the model description.

7. The deferral option should be defined more clearly. What is being deferred? The reader quickly realizes that the firm’s security investment (singular or plural?) may or may not be deferred. Can information sharing be deferred? Why does the reader have to search forever to find out whether or not information sharing can be deferred?

**Answer:** The deferral option is a strategy of firm’s security investment, not the strategy of firm’s information sharing. That is, firm’s cybersecurity investment can be deferred, firm’s information sharing cannot be deferred. All of these has been specified in the previous section of the model description.

8. The authors should ensure that all the articles in the reference list, checking one by one, are cited inside the article.

**Answer**: All the articles in the reference list has been checked. We have deleted one of the articles that is not cited inside the article.

9. The alpha is crucial, and should probably be different for the firm and the attacker.

**Answer:** $\alpha$ is different for the firm and the attacker, however, the effect of firm’s deferral investment on firm and attack’s expected benefits is positive correlation. Additionally, the difference of $\alpha$ between firm and attacker has no influence on our conclusion; hence, we assume the effect of firm’s deferral investment on the expected benefits of firm and attack is equal for the convenience of calculation. We have illustrated it in the Model Description.

10. The article’s focus on the deferral option pertains to whether the firm is proactive by investing early, or retroactive by investing later after a deferral. Comparison of the approach and results with the following article seems useful: Hausken, K. (2018), “Proactivity and Retroactivity of Firms and Information Sharing of Hackers,” International Game Theory Review 20, 1, 1750027, doi: 10.1142/S021919891750027X.

**Answer:** Similar to the proactive and retroactive defences in Hausken (2018), firm’s defence is proactive if it invests immediately, or retroactive if it invests later after a deferral.

The information sharing in this article is the information sharing between firms, but the information sharing in Hausken (2018) is the information sharing between hackers.

This paper investigates the effect of information sharing and deferral option on a firm’s expected benefits, and the firm and the attacker are simultaneously playing game. Notably, information sharing can improve the effect of deferral decision on a firm’s expected benefits when the accuracy of information sharing is low but weaken the effect when the accuracy of information sharing is high. Hausken (2018) analysis the interplay between the information sharing of hackers and the defense strategies of firms, and the game is four-period games. Notably, firm prefers to deter the first disadvantaged hacker when the two hackers benefit substantially from information sharing, reputation gain, or the second player is advantaged. All of these has been specified in the third and fourth paragraph of introduction.

11. More generally, comparing the approach and results with the articles in the reference list should be made more thoroughly, accounting for the fact that the article considers only one firm.

**Answer:** this article considers only one firm; however, this article assume the firm has joined an industry-specific information-sharing group, and there is no charges are incurred for joining this information-sharing group, providing that a firm is willing to share cybersecurity-related information with the group’s members (i.e., free-riders are excluded from this group). The relationship between firms is not considered, but the firm can share information with other firms in the group. Therefore, the results in this paper can be applied to the condition with two or more firms.

12. The weak abstract should be strengthened substantially, listing and discussing the results, emphasizing the contribution relative to the literature, etc. Conclusions can be written without parameters.

**Answer**: we have added the contribution of this paper relative to the literature ate the end of the abstract. The parameters in the conclusions are replaced with the definition.

Finally, we have employed a professional scientific editing service (Editage) for this study’s language usage, spelling, and grammar. We deeply appreciate your consideration of our manuscript, and give us good reviews. If you have any queries, please do not hesitate to contact us.

Thank you and best regards.

Yours sincerely

Chuanxi Cai
