# Supplementary material for: Information sharing and deferral option in cybersecurity investment
Source: PLoS One. 2023 Feb 6;18(2):e0281314. doi: 10.1371/journal.pone.0281314 (PMC9901811; doi:10.1371/journal.pone.0281314)
Supplement: Attachment — Submitted filename: Response to Reviewers1.docx [file pone.0281314.s004.docx]

Response to Reviewers

Dear Reviewers:

Thank you for your review, we have amended this article according to your advice. All the question and answer are as follows:

1. The authors have addressed my concerns, but ignored my request for a native reader. For example, the authors do not know when to use “the” and “a”, mixes past tense and present tense in the literature review, several typos exist, and the article ignores rules on singular/plural. For example, after equation (6) the following sentence is presented: “Fig. 3 indicate the game model.” Since Fig. 3 is singular, it should be “Fig. 3 indicates the game model.” or, which is better, “Fig. 3 presents the game model.”

**Answer:** we have checked and fixed all the rules on “the” and “a”, past tense and present tense, singular/plural. Additionally, several typos are also fixed. We deeply appreciate your consideration of our manuscript, and give us good reviews. If you have any queries, please do not hesitate to contact us.

Thank you and best regards.

Yours sincerely

Chuanxi Cai
